# 

**Published:** 1875-08

**Authors:** 


☞ All communications must be addressed to Dr. Thos. S. Powell, 82 Pryor street.

☞ Write your name, post-office, county and State *plainly*. Be sure to say to which post-office you wish the RECORD sent, and always date your letters. We receive many well written letters, but doubt even the authors being able to decipher their signatures.

☞ Send money by Check, Postal Order, or Registered Letter. We are willing to endorse for the honesty of our subscribers, but cannot be responsible for the irregularity of the mails.

☞ We hope our friends, one and all, will rally to the support of the RECORD. Send the publishers three dollars, the subscription fee, and at least one new subscriber. Send to the principal editor your communications, plainly and carefully written on only one side of the paper.

☞ All dues for 1873 and 1874 can be directed to Dr. T. S. Powell, or Powell & Goldsmith, as heretofore.

### COLLABORATORS AND CONTRIBUTORS.

GEORGIA.

James A. Long, M.D.
W. L. M. Harris, M.D.
H. L Battle, M.D.
W. C. Musgrove, M D.
L. B. Bouchelle, M.D.
Thomas Burdell, M.D.
E. H. W. Hunter, M.D.
V. S Hitt, M.D.
J. E. Pope, M.D.
J. C. Wisdom, M.D.
W. W. Leake, M.D.
S. G. White, M.D.
W. H. Hall, M.D.
G. D. Case, M.D.
W. T. Grant, M.D.
J. M. Tules, M.D.
J. N. Cheney, M.D.
T. M. Shaw, M.D.
T. S. Mitchell, M.D.
H. B. Lee, M.D.
C. B. Nottingham, M.D.
W. L. Kirkpatrick, M.D.

KENTUCKY.

W. S. Taylor, M.D.
B. W. Stone, M.D.
Charles Kearns, M.D.

TEXAS.

S. M. Welch, M.D.
T. J. Heard, M.D.
L. R. Lincecum, M.D.

TENNESSEE.

J. D. Tucker, M.D.
F. A. Ramsey, M.D.
J. W. Hayne, M.D.
F. K. Bailey, M.D.

WEST VIRGINIA.

N. D. Tobey, M.D.

ALABAMA.

J. C. Knox, M.D.
Geo. F. Taylor, M.D.
J. B. Barnett, M.D.
S. M. Hogan, M.D.
J. T. Searcy, M.D.
A. C. Edwards, M.D.
M. W. Morton, M.D.
J. D. Rush, M.D.
J. J. Grace, M.D.

NORTH CAROLINA.

J. B. Hughes, M.D.
S. S. Satchwell, M.D.
A. B. Pierce, M.D.
H. Kelly, M.D.
Geo. A. Foote, M.D.
E. A. Anderson, M.D.
H. T. Bahnson, M.D.
John W. Booth, M.D.
R. S. Hallsey, M.D.

SOUTH CAROLINA.

E. T. McSwain, M.D.
G. M. Rivers, M.D.

OHIO.

J. Q. A. Hudson, M.D.
C. H. Tidd, M.D.
J. C. Bishop, M.D.
W. P. Wells, M.D.

NEW YORK.

Ely Van DeWarker, M. D.
C. C. F. Gay, M.D.
J. S. Bailey, M.D.

COLORADO.

Wm. R. Whitehead, M.D.

VIRGINIA.

F. Horner, Jr., M.D.
R. J. Preston, M.D.
J. S. Wharton, M.D.
Sam'l P. Ripley, M.D.
E. A. Drewry, M.D.
Rob B. Tunstall, M.D.
W. F. Barr, M.D.
L. B. Anderson, M.D.
J. N. Upshur, M.D.
B. P. Reese, M.D.

MISSISSIPPI.

C. B. Gallaway, M.D.
Edward Lea, M.D.
S. V. D. Hill, M.D.
E. T. Henry, M.D.
T. P. Lockwood, M.D.
Robert Kells, M.D.
W. L. Lipscomb, M.D.
W. T. Beall, M.D.
A. G. Smythe, M.D.
J. M. Lewis, M.D.
J. H. Payne, M.D.
T. H. Mayo, M.D.
E. Cutter, M.D.

ARKANSAS.

A. D. Hodge, M.D.

J. K. Hodge, M.D.

A. W. Hobson, M.D.

C. D. Hodge, M.D.

INDIANA.

G. A. Dyer, M.D.
A. Patton, M.D.
Robert Robson, M.D.

ILLINOIS.

John H. Weir, M.D.

OREGON,

W. M. Cusick, M.D.

